# Interaction between cognitive, emotional and behavioral factors of sleep-related complaints in the normative sample

**DOI:** 10.1192/j.eurpsy.2022.2095

**Published:** 2022-09-01

**Authors:** E. Rasskazova

**Affiliations:** 1 Mental Health Research Center, Medical Psychology, Moscow, Russian Federation; 2 Moscow State University, Clinical Psychology, Moscow, Russian Federation

**Keywords:** psychological factors, sleep-related complaints

## Abstract

**Introduction:**

Complaints about sleep and sleepiness are widespread and are closely associated with dysfunctional beliefs about sleep, disturbed sleep hygiene and anxiety-depressive experiences (Perlis et al., 2011, Riemann et al., 2017, Sateia et al., 2017), however, the specific role and interactions of these factors are understudied.

**Objectives:**

The aim was to reveal the relationship between cognitive, emotional and behavioral factors of subjective sleep quality, sleepiness and typical patterns of nighttime sleep in the normative sample.

**Methods:**

224 people 18-47 years old without diagnosed sleep disorders answered questions about their sleep patterns, filled in the Insomnia Severity Index, Dysfunctional Beliefs About Sleep Scale (Morin, 1993), Behavioral Factors of Sleep Disorders Scale (Rasskazova, 2020), Epworth Sleepiness Scale (Johns, 1991), Glasgow Thought Content Inventory (Harvey, Espie, 2004), Hospital Anxiety and Depression Scale (Zigmond, Snaith, 1983).

**Results:**

The poorer subjective quality of sleep is predicted by more dysfunctional beliefs about sleep, cognitive arousal and disturbed sleep hygiene (R^2^=45.1%). The negative effect of cognitive arousal on sleep quality is higher in people with sleep hygiene disturbances (ΔR^2^=1.4%, p<.05). Only disturbance of sleep hygiene is a predictor of sleep duration, sleepiness and the experience of insufficient sleep (R^2^=9.9%-12.2%), while cognitive arousal (R^2^=23.4%) and (in people with higher sleep hygiene disturbances, ΔR^2^=3.5%, p<.01) negative emotions predict poorer sleep efficacy.

**Conclusions:**

Both relationship between cognitive arousal and poorer subjective sleep and relationship between anxiety, depression and poorer sleep efficacy are stronger in people with poorer sleep hygiene. Research is supported by the Russian Foundation for Basic Research, project No. 20-013-00740.

**Disclosure:**

Research is supported by the Russian Foundation for Basic Research, project No. 20-013-00740

